# Predictive factors of medium-giant coronary artery aneurysms in Kawasaki disease

**DOI:** 10.1038/s41390-023-02798-6

**Published:** 2023-09-05

**Authors:** Saitong Jiang, Meng Li, Kun Xu, Ying Xie, Piaohong Liang, Cong Liu, Qiru Su, Boning Li

**Affiliations:** 1 Shantou University Medical College, Shantou, Guangdong China; 2 Department of Cardiology, Shenzhen Children’s Hospital, Shenzhen, Guangdong China; 3 Institute of Pediatrics, Shenzhen Children’s Hospital, Shenzhen, Guangdong China

## Abstract

**Background:**

We aimed to examine predictive measures for medium and giant coronary artery aneurysms (CAA) in Kawasaki disease (KD) patients.

**Methods:**

Patients who were diagnosed with KD from 2015 to 2021 were retrospectively reviewed. The clinical and laboratory data were compared between medium-giant group and non-medium-giant group.

**Results:**

A total of 1331 KD patients were investigated, of whom 63 patients (4.7%) developed medium-giant CAA including 27 patients (2%) with giant CAA. Sex, age, fever duration, intravenous immunoglobulin (IVIG) resistance, platelet count, and albumin level independently predicted medium or giant CAA by multivariate logistic regression analysis. Male, age, duration of fever, IVIG resistance, platelet count, hemoglobin, and erythrocyte sedimentation rate were independent predictors for giant CAA. The two new scoring systems using these factors in identifying patients with medium-giant CAA and giant CAA had respectively sensitivities of 86.89% and 92.59%, and specificities of 81.65% and 87.93%. Validation in 2021 dataset (193 KD patients) showed comparable sensitivity and specificity to development dataset.

**Conclusions:**

Male, age, fever duration, IVIG resistance, platelet count, albumin, hemoglobin, and erythrocyte sedimentation rate might be significant predictors of medium and giant CAA. The sensitivity and specificity in our risk prediction model were higher than in previous research.

**Impact:**

- This is the first study to search for risk factors and establish a prediction model for the development of medium-giant CAA in the Chinese population using z-scores and absolute inner diameter values based on large sample sizes.
- The sensitivity and specificity in our model were higher than in previous studies.
- Our research could help clinicians better predict medium-giant CAA and choose more appropriate treatment.

## Introduction

Kawasaki disease (KD) is an acute systemic vasculitis that primarily affects small and medium arteries of children under the age of 5 years. Children with KD who develop coronary artery aneurysms (CAA) are more likely to contract acquired heart disease. With the progress of diagnosis and treatment of KD, the risk of coronary artery damage has been reduced, nonetheless, about 8% of patients still have cardiovascular complications.^1^

Coronary artery lesions can be divided into mild dilation and coronary aneurysms. According to the diameter of dilated coronary arteries, coronary aneurysms can be classified into small, medium, and large (or giant) aneurysms. Studies have shown that almost all small CAA (z scores of 2.5–5) can regress in the acute phase, while most medium and giant CAA without prompt intervention may persist and lead to myocardial infarction, blood vessel rupture, and even sudden death.^2–4^ Among them, giant CAA had the worst prognosis without significant change in maximum diameter after 1 year.^5^ Due to the unknown etiology, CAA in KD is hard to predict. Therefore, to decrease morbidity and mortality in children with KD, it is necessary to identify the risk factors that contribute to the susceptibility to mild to giant-size CAA development. There are a few studies identifying risk factors for the development of medium and giant coronary artery CAA but without risk prediction models. As a result, the present study analyzed the clinical data of children with KD to identify the risk factors for medium and giant CAA and to develop valuable risk prediction models.

## Methods

### Study design and population

A retrospective study was conducted on patients diagnosed with KD between January 2015 and December 2020 at Shenzhen Children’s Hospital. These data (development dataset) were used to develop a predictive model (*n* = 1331, medium or giant CAA = 63, giant CAA = 27). From January 2021 to December 2021, an additional 193 patients were treated and studied prospectively to test the accuracy of prediction (*n* = 193, medium or giant CAA = 17, giant CAA = 7). These data represented the validation dataset.

KD can be diagnosed as complete or incomplete based on the KD Research Committee’s 6th revised edition in Japan in 2020.^6^ Patients were excluded if they had received intravenous immunoglobulin (IVIG) or glucocorticoid treatment before admission, or had infectious diseases were excluded.

All patients were initially treated with IVIG 2 g/kg as a single infusion, given over 10–12 h, following American Heart Association Guidelines. Patients with persistent or recrudescent fever (≥38.0 °C) for at least 36 h but no longer than 7 days after receiving the first IVIG infusion were considered IVIG resistance.^7^ There were 1524 children enrolled in this study, including 956 boys and 568 girls, ranging in age from 2 to 116 months. All patients with KD who did not develop medium or giant CAA were compared with those who did. Shenzhen Children Hospital’s Ethics Committee approved this study.

### Data collection

The medical records were reviewed for demographics, clinical, laboratory, and treatment data. Demographics data includes sex, age (in months), clinical data includes diagnosis of incomplete KD, duration of fever before IVIG, total duration of fever, treatment data including whether receiving steroid therapy in initial therapy, and laboratory data before IVIG treatment include high-sensitivity C-reactive protein (CRP), neutrophils ratio, platelet count, white blood cell, hemoglobin, procalcitonin, erythrocyte sedimentation rate, alanine transaminase, aspartate aminotransferase, total bilirubin, albumin, serum sodium, and ferroprotein.

By echocardiography during hospitalization, we measured the maximum coronary arterial internal diameter of the right coronary artery, left main coronary artery, left anterior descending artery, and left circumflex coronary artery. With the help of a model based on data from a large cohort of Chinese healthy children, the maximum internal diameter of each artery in the coronary arteries was converted to a z-score corrected by the body surface area.^8^ In addition, we followed up the regression rate of medium and giant CAA 1 year after diagnosis. Medium CAA was defined as a z-score of ≥5–<10, or an absolute dimension of ≥4–<8 mm, and giant CAA as a z-score of ≥10, or an absolute dimension of ≥8 mm.^1^ CAA was considered to have regressed when the enlarged coronary arteries had normal z score or dilation only (z-score of <2.5) as visualized by echocardiogram.

### Statistics analysis

Statistical analyses were conducted using Stata MP 17.0. Categorical variables were described by frequency and constituent ratio, and chi-square tests were used to compare differences between groups. A median with a quartile range is reported for continuous variables, and the difference between the two groups is compared using an independent sample T-test. To identify independent risk factors and develop risk prediction models, logistic regression analysis was used with a forward stepwise method.

The regression model was tested for fitness with the Hosmer–Lemeshow test. To evaluate the model’s capacity, the area under the receiver operating characteristic (ROC) curve (AUC) was calculated, and the cutoff values were determined by the Youden index derived from the calculated sensitivity and specificity. Ten folds cross-validation test was adopted in this study to validate the model’s prediction accuracy. The data from January 2021 to December 2021 were used for external verification. Additionally, we compared AUCs between logistic regression and scoring models. *P* < 0.05 was considered significantly different.

We assigned points to each risk predictor to develop an easy-to-use scoring model. Scores for each predictor in the scoring system were calculated based on the corresponding model coefficients and were re-scaled between 0 to 10 points using the following steps. First, the highest original score for each predictor was determined. A score for each predictor was calculated by multiplying its coefficients with its maximum value. We assigned a maximum score of 10 points to the prediction with the highest possible score, and we rescaled all other predictions using this same scaling factor. Based on the highest and lowest re-scaled scores for each predictor, the increase in the re-scaled score per one unit of the predictor value was calculated to obtain the equations for calculating an individual’s total score. To define the threshold for the scoring system, the model thresholds were transformed into scores. The starting value for each model was then calculated based on the minimum value of each predictor (or the maximum value of predictors with negative coefficients) and the model intercept. The original score at the threshold for the medium and giant CAA model was then calculated and distinguished between the high and low risk of CAA. Supplementary Appendixes A and B contain information about these transformations as well as a worked-out example.

## Results

### Baseline characteristics

This study included 1331 children (826 males and 505 females) who were hospitalized for KD. The median age was 22 months (ranging from 2 months to 9.7 years). 141 cases (10.6%) were diagnosed with incomplete KD. The median days of fever before IVIG was 6 days (ranged from 3 days to 24 days) and 258 patients (19.4%) received initial treatment after seven days of illness. 92 patients (6.9%) who met the indication of the initial steroid therapy received IVIG combined with systemic glucocorticoids in the first treatment after being diagnosed with Kawasaki disease. The total duration of fever ranged from 3 days to 26 days. Among them, 108 (8.1%) were defined as IVIG non-responders, and 63 (4.7%) were complicated by medium or giant coronary aneurysms (36 medium coronary aneurysms and 27 giant coronary aneurysms). Of the 63 patients with medium or giant coronary aneurysms, 27(42.9%) were irresponsive to the first dose of IVIG, and 5 cases (7.9%) were co-administered with IVIG and systemic glucocorticoids. In comparison, 13(48.1%) out of 27 patients with giant coronary aneurysms were irresponsive to the first dose of IVIG, and 4 cases (14.8%) were co-administered with IVIG and systemic glucocorticoids. The regression rates 1 year after diagnosis were only 9.7% for medium CAA (5 patients were lost to follow-up) and none of giant CAA (1 patient was lost to follow-up) regressed to normal z score or dilation only after 1 year.

### Comparison of clinical characteristics between the medium-giant CAA and non-medium-giant CAA groups

Males made up 50 (79.4%) of the group with medium or giant CAA and 776 (61.2%) of the group without it (*P* < 0.05). In the medium or giant CAA group, the age ranged from 2 to 116 months, with a median age of 27 months, whereas in the non-medium or giant CAA group, the age ranged from 2 to 107 months, with a median age of 22 months (*P* > 0.05). Duration of fever before IVIG ranged from 5 to 13 days, with a median duration of 7 days, in the medium or giant CAA group, whereas in the non-medium or giant CAA group, the duration ranged from 5 to 7 days, with a median duration of 6 days. The prevalence of incomplete KD and IVIG resistance, the days of fever before IVIG and the total duration of fever differed substantially (*P* < 0.05) between the two groups (Table 1). In addition, the initial therapy with IVIG and systemic glucocorticoids in the medium or giant CAA group and non-medium or giant CAA group were respectively in 6.9% and 7.9% of patients, which showed no statistical significance (*p* > 0.05).

**Table 1:** Comparison of clinical characteristics between the medium or giant CAA and non-medium or giant CAA groups.

	Training set			Validation set	
	Kawasaki disease patients admitted in Shenzhen Children’s Hospital from January 2015 to December 2020 (*n* = 1331)			Kawasaki disease patients admitted in Shenzhen Children’s Hospital from January 2021 to December 2021 (*n* = 193)	
Variables	Medium or giant CAA (*n* = 63)	Non-medium or giant CAA (*n* = 1268)	*P* value	Medium or giant CAA (*n* = 17)	Non-medium or giant CAA (*n* = 176)
Male, *N*(%)	50(79.4)	776(61.2)	0.004	14(82.4)	116(65.9)
Age, months	27(13, 47)	22(12, 37)	0.055	14.0(6.5, 35.0)	23.00(16.00, 41.75)
Incomplete KD, *N*(%)	16(25.4)	125(9.9)	0.000	3(17.6)	6(3.4)
Duration of fever before IVIGs, days	7(5, 13)	6(5, 7)	0.000	7(4, 11)	5(5, 6)
Total duration of fever, days	10(7, 14)	6(5, 8)	0.000	12.0(7.0, 17.5)	6(5, 7)
IVIG resistance, *N*(%)	27(42.9)	81(6.4)	0.000	10(58.8)	12(6.8)
Initial therapy combined with GCS, *N*(%)	5(7.9)	87(6.9)	0.743	6(35.3)	33(18.8)
High-sensitivity C-reactive protein, mg/L	66.67(44.80, 116.80)	63.00(36.40, 100.98)	0.118	72.04(36.43, 119.99)	57.60(33.50, 89.53)
Neutrophils ratio, %	71.6(55.9, 81.3)	67.45(57.48, 77.03)	0.368	62.80(56.45, 72.80)	69.80(61.18, 79.18)
Platelet count, 10^9^/L	487(332, 681)	376(311, 457)	0.000	418(306, 607)	358(297, 428)
White blood cell, 10^9^/L	15.12(11.67, 18.43)	14.68(11.81, 18.49)	0.402	12.80(8.15, 17.40)	14.12(11.79, 17.42)
Hemoglobin, g/L	104(96, 109)	109(103, 115)	0.000	105(90, 113)	111(105, 116)
Procalcitonin, ng/ml	0.32(0.10, 1.27)	0.46(0.15, 1.40)	0.287	0.58(0.22, 3.70)	0.85(0.31, 2.51)
Erythrocyte sedimentation rate, mm/h	75.00 (54.00, 97.25)	68(48, 87)	0.042	49.50(42.00, 78.25)	61(46, 79)
Aspartate aminotransferase, IU/L	30.0 (22.0, 47.5)	30(23, 45)	0.300	40.0(31.5, 70.5)	29(23, 39)
Alanine transaminase, IU/L	25.50(12.50, 91.75)	32.0(15.5, 84.5)	0.786	31.0(18.5, 100.0)	31.5(16.0, 77.0)
Total bilirubin, μmol/L	6.80(3.93, 13.35)	7.1(5.0, 10.2)	0.016	7.40(3.45, 12.75)	7.1(5.0, 9.6)
Albumin, g/L	33.70(31.10, 35.75)	35.60(32.9, 37.98)	0.000	33.3(29.7, 36.6)	36.70(34.65, 38.93)
Serum sodium, mmol/L	135.10(132.80, 137.05)	135.1(133.2, 136.8)	0.535	135.50(132.85, 136.70)	134.95(132.78, 136.33)
Ferroprotein, ng/ml	167.92(128.60, 261.20)	151.04(105.39, 220.20)	0.199	228.49(161.74, 348.01)	169.26(127.01, 249.04)

In comparison to the non-medium and giant CAA group for laboratory parameters, platelet count and erythrocyte sedimentation rate were significantly higher (*P* < 0.05) in the medium or giant CAA group. The levels of hemoglobin, albumin and total bilirubin levels in the medium or giant CAA group were significantly lower than in the non-medium or giant CAA group (*P* < 0.05) (Table 1).

### Comparison of clinical characteristics between the giant CAA and non-giant CAA groups

There were respectively 805 (61.7%) males and 21 (77.8%) males in the non-giant CAA group and giant CAA group. In the giant CAA group, the age varied from 3 to 116 months, with a median age of 30 months, whereas in the non-giant CAA group, the age ranged from 2 to 107 months, with a median age of 22 months. Duration of fever before IVIG ranged from 5 to 14 days, with a median duration of 8 days, in the giant CAA group, whereas in the non-giant CAA group, the duration ranged from 5 to 7 days, with a median duration of 6 days. Significant differences (*P* < 0.05) were demonstrated in the frequency of IVIG resistance, the days of fever before IVIG, and the total duration of fever between the two groups (Table 2). Moreover, 14.8% of patients received IVIG combined with systemic glucocorticoids in their initial therapy in the giant CAA group, while 6.7% were in the non-giant CAA group, which were no statistical significance.

**Table 2:** Comparison of clinical characteristics between the giant CAA and non-giant CAA groups.

	Training set			Validation set	
	Kawasaki disease patients admitted in Shenzhen Children’s Hospital from January 2015 to December 2020 (*n* = 1331)			Kawasaki disease patients admitted in Shenzhen Children’s Hospital from January 2021 to December 2021 (*n* = 193)	
Variables	Giant CAA (*n* = 27)	Non-giant CAA (*n* = 1304)	*P* value	Giant CAA (*n* = 7)	Non-giant CAA (*n* = 186)
Male, *N*(%)	21 (77.8)	805 (61.7)	0.089	7 (100.0)	123 (66.1)
Age, months	30 (17, 54)	22 (12, 38)	0.046	17 (11, 52)	23(14, 41)
Incomplete KD, *N*(%)	5 (18.5)	136 (10.4)	0.176	1 (14.3)	8(4.3)
Duration of fever before IVIGs, days	8 (5, 14)	6(5, 7)	0.002	9 (4, 11)	5 (5, 6)
Total duration of fever, days	10 (8, 14)	6 (5, 8)	0.000	12 (7, 23)	6 (5,7)
IVIG resistance, *N*(%)	13 (48.1)	95 (7.3)	0.000	5 (71.4)	17(9.1)
Initial therapy combined with GCS, *N*(%)	4 (14.8)	88 (6.7)	0.210	1 (14.3)	38 (20.4)
High-sensitivity C-reactive protein, mg/L	74.62 (56.70, 145.79)	63.0 (36.4, 101.0)	0.024	57.42 (31.48, 184.90)	58.46 (34.70, 91.69)
Neutrophils ratio, %	71.6 (57.4,84.1)	67.50 (57.35, 77.10)	0.312	59.1 (54.7, 71.4)	69.80 (60.95, 78.35)
Platelet count, 10^9^/L	532 (304, 698)	377 (312, 459)	0.043	418 (367, 673)	358.0 (296.5, 432.5)
White blood cell, 10^9^/L	14.84 (12.39, 18.43)	14.70 (11.80, 18.49)	0.369	12.80 (7.74, 17.32)	14.07 (11.73, 17.44)
Hemoglobin, g/L	103 (95, 109)	109.00 (102.75, 115.00)	0.001	93 (87, 108)	111.0 (104.5, 116.0)
Procalcitonin, ng/ml	0.25(0.11, 1.22)	0.46 (0.15, 1.41)	0.363	0.54 (0.18, 12.32)	0.86 (0.29, 2.64)
Erythrocyte sedimentation rate, mm/h	93 (74, 104)	68 (48, 87)	0.000	75.50 (45.75, 102.00)	60.0 (45.5, 78.5)
Aspartate aminotransferase, IU/L	26 (22, 43)	30 (23, 45)	0.618	33 (29, 77)	29.0 (23.0, 40.5)
Alanine transaminase, IU/L	25 (12, 78)	31.5 (15.0, 85.25)	0.688	20 (17, 86)	31.5 (16.0, 79.0)
Total bilirubin, μmol/L	6.3 (4.1, 32.2)	7.1 (5.0, 10.3)	0.056	6.8 (3.3, 35.2)	7.2 (4.9, 9.6)
Albumin, g/L	33.4 (29.4, 35.2)	35.5 (32.8, 37.9)	0.000	31.0 (25.6, 33.4)	36.65 (34.43, 38.85)
Serum sodium, mmol/L	135.4 (132.1, 137.8)	135.1 (133.2, 136.8)	0.529	133.5 (132.6, 137.3)	135.0 (132.8, 136.3)
Ferroprotein, ng/ml	175.68 (138.04, 286.35)	151.38 (105.17, 220.80)	0.143	241.32 (164.46, 296.03)	179.26 (129.62, 259.03)

As for laboratory parameters, the levels of CRP, platelet count, erythrocyte sedimentation rate, and in the giant CAA group were significantly higher than those in the non-giant CAA group (*P* < 0.05). The levels of hemoglobin and albumin in the giant CAA group were significantly lower than in the non-giant CAA group (*P* < 0.05) (Table 2).

### Risk prediction model for medium or giant coronary aneurysms

**Fig. 1:**
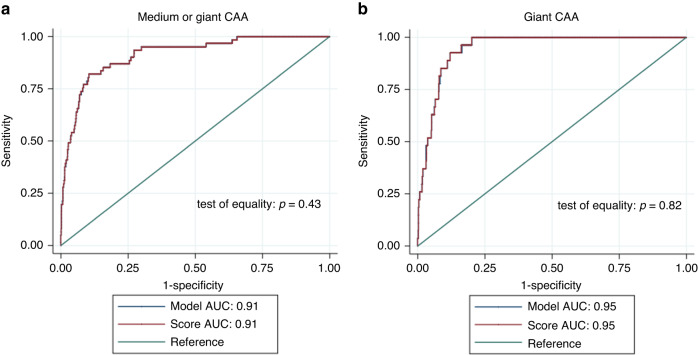
Receiver operating characteristic (ROC) curve of the logistic regression model and the scoring system for medium or giant CAA. In the logistic regression model and the scoring system, cut-off values of −0.0398 and 8.92 points yielded sensitivities of 86.89% and 86.89%, and specificities of 81.74% and 81.65%, respectively. The area under the curve (AUC) was 0.91 both in the logistic regression model and the scoring system. The comparison between the model’s and score’s AUCs showed no statistical difference (*P* = 0.43) (**a**); ROC curve of the logistic regression model and the scoring system for giant CAA. In the logistic regression model and the scoring system, cut-off values of −0.0289 and 15.34 points both yielded sensitivities of 92.59% and specificities of 87.93%, respectively. The AUC was 0.95 both in the logistic regression model and the scoring system. The comparison between the model’s and score’s AUCs showed no statistical difference (*P* = 0.82) (**b**).

Twenty clinical and laboratory factors (male, age, incomplete KD, duration of fever before IVIG, total duration of fever, IVIG resistance, initial therapy combined with glucocorticoids, CRP, neutrophils ratio, platelet count, white blood cell, hemoglobin, procalcitonin, erythrocyte sedimentation rate, alanine transaminase, aspartate aminotransferase, total bilirubin, albumin, serum sodium, ferroprotein) were analyzed using logistic regression. Male, duration of fever, IVIG resistance, platelet count, and albumin were independent predictors of medium or giant CAA (Table 3). The Hosmer–Lemeshow value was 0.706, which showed that the observed event rate and the model did not deviate significantly. Cut-off values of 0.0398 in the logistic regression model resulted in a sensitivity of 86.89% and a specificity of 81.74%, respectively. This regression model’s area under the ROC curve was 0.913 (95% confidence interval (CI) 0.877, 0.950) (Fig. 1a).

**Table 3:** Independent factors identified by multiple logistic regression analysis for predicting medium or giant CAA.

Variables	Logistic coefficient(β)	Odd ratio (95% CI)	*P* value
Male	1.121	3.068 (1.469, 6.410)	0.003
Age	0.027	1.027 (1.014, 1.041)	0.000
Total duration of fever	0.222	1.248 (1.149, 1.357)	0.000
IVIG resistance	2.323	10.208 (4.664, 22.342)	0.000
PLT, 10^9^/L	0.008	1.008 (1.005, 1.010)	0.001
ALB, g/L	−0.134	0.875 (0.806, 0.950)	0.000

*CI* confidence interval, *PLT* platelet count, *ALB* albumin.

The point for each risk predictor was assigned and a scoring model was obtained by the following calculation formula:

Medium or giant CAA score = Sex * 1.3182 + (Age − 2) * 0.0317 + (Total duration of fever − 3) * 0.2609 + IVIG resistance * 2.7315 + (PLT − 45) * 0.0088 + (ALB − 45.60) * (−0.1596)

Note: Sex: male = 1, female = 0; Age, months; Total duration of fever, days; IVIG resistance: Yes = 1, No = 0; PLT: platelet count, 109/L; ALB: albumin, g/L.

Using a cut-off of 8.97 points with this risk prediction score, KD patients with medium and giant CAA were with 86.89% sensitivity and 81.57% specificity. The AUC of this scoring system was 0.914 (95% CI 0.877, 0.950) (Fig. 1a). AUCs generated from the model and the scoring system for determining the risk of medium or giant CAA showed no significant difference (*p* > 0.05).

**Fig. 2:**
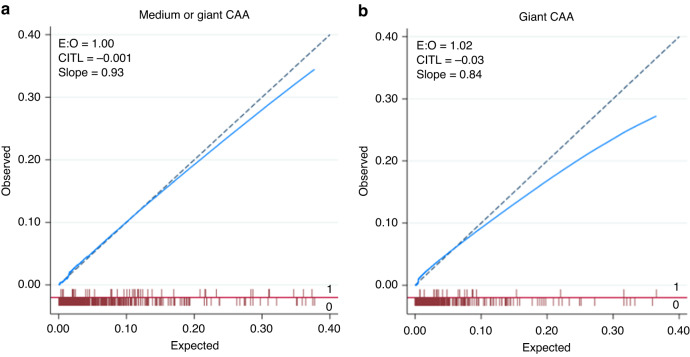
Calibration plot of the model predicting CAA. **a** Medium or giant CAA; **b** Giant CAA.

**Fig. 3:**
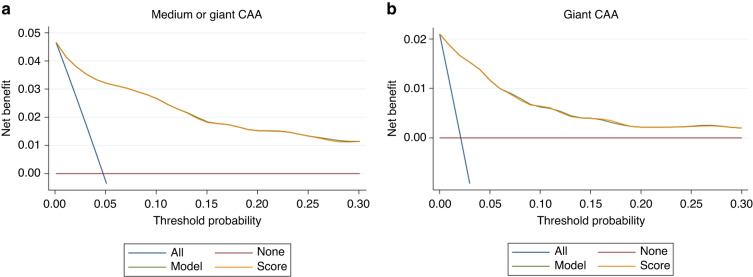
Decision curve analysis on the model predicting CAA. **a** The model and the scoring system for assessing the risk of medium or giant CAA; **b** The model and the scoring system for assessing the risk of giant CAA.

The 10-fold internal cross-validation was used to assess model performance, and discrimination was good (cross-validation (CV) AUC 0.904, 95% CI 0.863, 0.944). With a CV calibration-in-the-large (CITL) of 0.001 and a CV slope of 0.93, this definition was accurately calibrated to the reference standard (Fig. 2a). To assess the model’s and the scoring system’s clinical effectiveness, decision curve analysis (DCA) was used (Fig. 3a).

### Risk prediction model for giant coronary aneurysm

Twenty variables in Table 2 were evaluated by multivariate logistic regression. The results showed that age, male, duration of fever, IVIG resistance, platelet count, hemoglobin, and erythrocyte sedimentation rate were independent risk predictors of the giant coronary aneurysm (Table 4). The Hosmer–Lemeshow statistic for this regression model was 0.945, and the AUC was 0.948 (95% CI 0.927, 0.969) (Fig. 1b). Cut-off values of 0.0289 in the logistic regression model resulted in a sensitivity of 92.59% and a specificity of 87.93%.

**Table 4:** Independent factors identified by multiple logistic regression analysis for predicting giant CAA.

Variables	Logistic coefficient(β)	Odd ratio (95% CI)	*P* value
Male	1.127	3.086 (1.075, 8.853)	0.036
Age	0.030	1.031 (1.011, 1.050)	0.002
Total duration of fever	0.169	1.184 (1.071, 1.311)	0.001
IVIG resistance	2.310	10.073 (3.459, 29.334)	0.000
PLT, 10^9^/L	0.05	1.005 (1.002, 1.008)	0.001
HGB, g/L	−0.046	0.955 (0.915, 0.997)	0.037
ESR, mm/h	0.037	1.037 (1.014, 1.061)	0.001

*CI* confidence interval, *PLT* platelet count, *HGB* hemoglobin, *ESR* erythrocyte sedimentation rate.

A scoring model was obtained by the following calculation formula:

Giant CAA score = Sex * 2.0699 + (Age − 2) * 0.0553 + (Total duration of fever − 3) * 0.3110 + IVIG resistance * 4.2436 + (PLT − 45) * 0.0088 + (HGB − 146) * (−0.0838) + ESR * 0.0672

Note: Sex: male = 1, female = 0; age, months; Total duration of fever, days; IVIG resistance: Yes = 1, No = 0; PLT: platelet count, 10^9^/L; HGB: hemoglobin, g/L; ESR: erythrocyte sedimentation rate, mm/h.

Using a cut-off of 18.74 points with this risk prediction score, KD patients with giant CAA were with 92.59% sensitivity and 87.93% specificity. The AUC of this scoring system was 0.948 (95% CI 0.927, 0.969) (Fig. 1b). The AUCs generated from the model and the scoring system for determining the risk of giant CAA were not significantly different (*p* > 0.05).

Discrimination was good (CVAUC 0.931, 95% CI 0.903, 0.959) by the 10-fold internal cross-validation. With a CV CITL of −0.03 and a CV slope of 0.84, this definition was accurately calibrated to the reference standard (Fig. 2b). To assess the model’s and the scoring system’s clinical effectiveness, decision curve analysis (DCA) was used (Fig. 3b).

### Validation dataset

The validation dataset was utilized to evaluate how accurately the risk model identified medium and giant CAA. In the prospective data (from January 2021 to December 2021), there were medium or giant CAA accounted for 17 of 193 KD patients (8.8%) (Table 1). In the validation dataset, the AUC of the medium or giant CAA model was 0.906 (95% CI 0.813, 0.999). Sensitivity and specificity were 82.35% and 89.60%, respectively. In addition, giant CAA accounted for 7 of 193 KD patients in the validation dataset (3.6%) (Table 2). In the validation dataset, the AUC of the giant CAA model was 0.937 (95% CI: 0.852, 1.000). The sensitivity and specificity were, respectively, 83.33% and 91.06%.

## Discussion

Several scoring models for predicting the development of CAA have been established previously. However, these models have significant variations in predictive efficacy among different populations. Recently, some Japanese risk scores were applied to predict CAA risks in non-Japanese populations, but they did not accurately predict poor outcomes in non-Japanese populations.^9–11^ Therefore, we attempted to develop a new risk models to predict CAA in the Chinese population.

In this training and validation study, two risk prediction models for medium or giant coronary aneurysm and giant coronary aneurysm were developed. In addition, this study showed that male, age, total duration of fever, IVIG resistance, platelet count, and albumin were independent predictors for medium or giant coronary aneurysms. A logistic regression model with six indicators was created to predict KD in children with medium or giant CAA, and the cut-off of −0.0398 produced sensitivity and specificity values of 86.89% and 81.74%, respectively. The cut-off score of 8.92 points was established for use in clinical practice, and it produced 86.89% sensitivity and 81.65% specificity.

Independent risk factors of giant CAA were similar to medium or giant CAA risk factors. Among these factors, male, age, total duration of fever, IVIG resistance, platelet count, hemoglobin, and erythrocyte sedimentation rate were independent predictors of giant CAA. The cut-off of −0.0398 yielded sensitivity and specificity values of 86.79% and 81.74%, respectively, in a logistic regression model with six factors to predict KD in children with medium or giant CAA. 86.89% sensitivity and 81.65% specificity were achieved by the cut-off score of 8.92 points, which was created for use in clinical practice.

CAA is a serious complication of KD. Compared with small CAA, most medium and giant CAA persist which is an important cause of mortality in children with KD. To the best of our knowledge, this is the first study to search for risk factors and establish a prediction model for the development of medium-giant CAA in the Chinese population using z-scores and absolute inner diameter values. Mary Beth et al.^12^ analyzed data from 903 KD patients and indicated that left anterior descending or right coronary artery Z score ≥2.0, age <6 months, Asian ethnicity, and CRP ≥ 13 mg/dL may be the potential candidates for CAA predictors and constructed a risk prediction model for CAA of which AUC was 0.82. Liu et al.^10^ selected 203 KD patients to develop a scoring system comprised of five different predictors: days of disease at initial therapy ≥7, redness and edema of extremities, hematocrit ≤33%, proportion of monocytes ≥8.89%, and procalcitonin ≥0.5 ng/mL. The newly constructed scoring system has an AUC of 0.685, with a sensitivity of 41.18% and a specificity of 84.41%. To date, a few grading methods have been developed to estimate CAA hazards. These scoring methods, however, were not based on large sample sizes and had lower sensitivity and specificity than the risk prediction model produced in our study.

The prevalence of giant CAA was 2.2% in our study, which is higher than 0.1% reported in studies conducted by the nationwide KD survey in Japan during 2017–2018.^13^ This difference may be because we have information based on both the absolute dimension of coronary and Z-score evaluation. The adoption of the Z-score criterion may result in more CAA detection.^14,15^ On the other hand, the high incidence of giant CAA may also be related to the delay in receiving initial IVIG in our study. Nearly 20% of patients in our study received initial treatment after 7 days of illness, while a recent survey showed that nearly 90% of patients in Japan received initial IVIG therapy within 7 days of illness.^16^ Some studies showed that compared with conventional therapy (within 7 days), late IVIG therapy may have a higher risk for coronary aneurysms.^10,16^

Previous research on long-term coronary outcomes in KD patients with CAA found that larger-sized coronary lesions were less likely to regress.^3,5,17,18^ Advani et al.^3^ found that CAA with a maximum diameter of ≥6.0 mm or Z ≥ 7.5 at 2 months after KD persisted at 15 years after KD. In our study, the regression rate of medium and giant CAA was only 5.3% at 1 year after the diagnosis of KD. Therefore, identifying potential risk factors for the development of larger-sized coronary lesions plays an important role in the treatment of KD.

In our study, 8.6% of patients received glucocorticoids in their initial therapy. In China, the use of steroids in the initial therapy of KD is suitable for Kawasaki disease shock syndrome (KDSS), KD with macrophage activation syndrome (MAS), and patients at high risk for nonresponse to IVIG defined by scoring systems including Kobayashi score or risk scores for predicting nonresponse to IVIG assigned by various hospitals. For patients who are IVIG resistance, it is recommended that receiving retreatment with high dose of IVIG, giving corticosteroids, or using infliximab (IFX). Chinese clinicians usually apply methylprednisolone 2 mg/ (kg·d) and decreased the dose gradually until CRP is normal, or intravenous high-dose methylprednisolone 10–30 mg/(kg·d) for 3–5 days, followed by oral prednisone 2 mg/ (kg·d), and then progressively discontinued. The total course of treatment for corticosteroids will be more than 2 weeks.^19^

Each of the variables in our model has also been shown to be a CAA risk factor.^20–23^ Male constituted a higher risk for medium to large CAA and other studies had also found that patients with large CAA were almost exclusively male, suggesting that sex may have a key influence in coronary artery disease in KD.^20,22,24,25^ Younger age may influence IVIG response. Despite prompt identification and treatment, age at illness onset of fewer than 6 months is associated with a negative result.^21,26,27^ As a result, one of the elements in the Egami and Kobayashi score is the age at diagnosis.^28,29^ Delayed diagnosis, along with incomplete clinical manifestation, has been indicated to be the major contributor to the development of CAA.^22^ In the current study, although incomplete KD and duration of fever before IVIG were not the independent risk factors for CAA, our data suggested that they were associated with medium and giant CAA.

A prolonged fever raises the risk of medium and giant coronary aneurysms. As a consequence, the objective of therapy during the acute phase of KD is to shorten fever duration, reduce systemic inflammation, and avoid vascular damage.^30^

In the present study, the incidence of IVIG-resistant KD was 8.1%, which is similar to previously reported data.^31–33^ Numerous studies have shown that timely IVIG therapy reduces the development and severity of coronary vasculitis. As with previous studies, patients who did not response to IVIG therapy in this study were about 10 times more likely to develop medium and giant CAA than those who were sensitive to IVIG.

Platelet count, hemoglobin, albumin, and erythrocyte sedimentation rate could be easily obtained from the blood test and are widely proven to be useful prognostic indicators of KD. Although the precise mechanism of thrombocytosis is uncertain, it has been hypothesized that an acute inflammatory response mediated by high thrombopoietin levels may cause thrombocytosis.^34^ Previous research found that anemia of inflammation induced by impaired erythropoiesis and reduced iron availability in critically sick individuals was an independent predictor of higher mortality.^35,36^ Lower hemoglobin levels may reflect that greater underlying inflammation with a more intense systemic vasculitis and more profound myocardial involvement. Thus, lower hemoglobin levels in the acute period of KD may be a significant risk factor for CAA. Hypoalbuminemia was often reported in individuals with KD, which may have occurred mostly from enhanced microvascular permeability during the acute phase.^37^ In the present study, Lower albumin levels have been associated with the development of medium or giant CAA formation, which may be indicative of more inflammation and vascular leakage in coronary aneurysms. An elevated erythrocyte sedimentation rate was found in almost all children with KD, which was an important variable to predict IVIG resistance in previous studies,^38,39^ and we found erythrocyte sedimentation rate was a useful predictor of giant CAA, highlighting its role in exuberant inflammation in KD.

The most important complication associated with KD is the formation of medium and giant coronary aneurysms, which is the most significant issue affecting the prognosis. As a result, the primary treatment objective for KD patients is to avoid the development of medium and giant CAA. This study constructed two risk prediction models to predict KD patients at high risk of complicating with medium and giant CAA, especially for patients who did not respond to IVIG or had a long duration of fever. We recommend that such patients use this predictive formula for prediction and they may require more rigorous treatments and more careful follow-up of echocardiography during acute KD. Further long-term studies to assess the risks of developing medium and giant CAA with KD are needed.

## Limitations

There are some limitations to our study. First, this study was done at a single Chinese tertiary medical center, which might have resulted in selection bias. Second, this was a retrospective investigation, and thus, it is difficult to assess certain data except for the patient’s information in the medical record. Third, the evaluation of echocardiogram measurements was not centralized and standardized. Forth, coronary abnormalities on initial echocardiograms were not analyzed. Finally, other treatments like aspirin were not taken into consideration, which was potential confounders in the current study.

## Conclusions

Two risk prediction models to predict the occurrence of medium or giant CAA and predict giant CAA were developed and validated in the present study. Moreover, we found that male, age, total duration of fever, IVIG resistance, platelet count, and albumin were independently associated with medium or giant CAA formation. The scoring approach employed in this study to distinguish between groups with a low and high risk of CAA may be a useful tool for guiding more efficient initial therapy and echocardiographic follow-up. Future larger studies with a more diverse population in China are needed to test this risk score and to assess accuracy and generalizability.

### Supplementary information

Appendix A

Appendix B

## Data Availability

The datasets generated during and analyzed during the current study are available from the corresponding author upon reasonable request.
