# Supplementary material for: Predictive factors of medium-giant coronary artery aneurysms in Kawasaki disease
Source: Pediatr Res. 2023 Sep 5;95(1):267–74. doi: 10.1038/s41390-023-02798-6 (PMC10798897; doi:10.1038/s41390-023-02798-6)
Supplement: Supplementary file 1 — Appendix A [file 41390_2023_2798_MOESM1_ESM.pdf]

Scores for each predictor in the scoring system were calculated based on the corresponding model coefficients and were re-scaled between 0 to 10 points using the following steps:

1. The highest original score for each predictor was determined. For binary variables, the highest original score was directly determined by the coefficient, e.g., the coefficient of male sex for the medium or giant CAA model was 1.1211, the maximum value of male sex was 1, resulting in a highest original score of 1.1211. For continuous variables, the highest original score was determined by the maximum value of the variable, e.g., the maximum value of PLT was 1134, resulting in a highest original score of 0.0075*1134=8.5050 for the medium or giant CAA model.
2. For predictors with negative coefficients, the score was forced positive, and the maximum value was determined by the range in values. For example, ALB ranges from 22 to 46, then the maximum value was 22-46=-24, and the highest original score was -0.1335*-24=3.2040 for the medium or giant CAA model.
3. Among all highest original scores for the medium or giant CAA model, the score of PLT was the highest (8.5050). A score of 10 points was then assigned to a PLT value of 1134. This corresponds to a re-scaling factor of 10/8.5050=1.1758. Repeating the above steps for the giant CAA model obtained a re-scaling factor of 1.8372.

4. Other original scores were re-scaled by multiplying the re-scaling factors for the two models, respectively. The results were presented in the following table.

	Coefficient	Minimum	Maximum	Highest score	lowest score	Re-scaled highest score	Re-scaled lowest score	Increase in the re-scaled score/unit
**Medium or giant CAA**								
Sex	1.1211	0	1	1.1211	0.0000	1.3182	0.0000	1.3182
Age	0.027	2	116	3.1320	0.0540	3.6825	0.0635	0.0317
Total duration of fever	0.2219	3	26	5.7694	0.6657	6.7835	0.7827	0.2609
IVIG resistance	2.3231	0	1	2.3231	0.0000	2.7315	0.0000	2.7315
PLT	0.0075	45	1134	8.5050	0.3375	10.0000	0.3968	0.0088
ALB	-0.1335	22	46	3.2040	0.0000	3.7672	0.0000	-0.1596
**Giant CAA**								
Sex	1.1267	0	1	1.1267	0.0000	2.0699	0.0000	2.0699
Age	0.0301	2	116	3.4916	0.0602	6.4146	0.1106	0.0553

Total duration of fever	0.1693	3	26	4.4018	0.5079	8.0868	0.9331	0.3110
IVIG resistance	2.3099	0	1	2.3099	0.0000	4.2436	0.0000	4.2436
PLT	0.0048	45	1134	5.4432	0.2160	10.0000	0.3968	0.0088
HGB	-0.0456	75	146	3.2376	0.0000	5.9480	0.0000	-0.0838
ESR	0.0366	0	122	4.4652	0.0000	8.2033	0.0000	0.0672

Note: Sex: male=1, female =0; Age, months; Total duration of fever, days; IVIG resistance: Yes=1, No=0; PLT: platelet count, 10^9/L; ALB: albumin, g/L; HGB: hemoglobin, g/L; ESR: erythrocyte sedimentation rate, mm/h

5. Based on the highest and lowest re-scaled scores for each predictor, the increase in the re-scaled score per one unit of the predictor value (see the above table) were calculated to obtain the equations for calculating an individual's total score (showed as the following).

- Medium or giant CAA total score = sex*1.3182+ (age - 2) *0.0317+ (total duration of fever - 3) *0.2609+ IVIG resistance*2.7315+ (PLT - 45) *0.0088+ (ALB - 45.60) *(-0.1596)

  Note: sex: male=1, female =0; age, months; total duration of fever, days; IVIG resistance: Yes=1, No=0; PLT: platelet count, 10^9/L; ALB: albumin, g/L

- Giant CAA total score = sex*2.0699+ (age - 2)*0.0553+ (total duration of fever - 3)*0.3110+ IVIG resistance*4.2436+ (PLT - 45)*0.0088+ (HGB – 146)*(-0.0838) + ESR*0.0672

  Note: sex: male=1, female =0; age, months; total duration of fever, days; IVIG resistance: Yes=1, No=0; PLT: platelet count, 10^9/L; HGB: hemoglobin, g/L; ESR: erythrocyte sedimentation rate, mm/h

6. To define the threshold for the score system, the model thresholds (3.98% for the medium or giant CAA model and 2.89% for the giant CAA model) were transformed to scores. The linear predictor was calculated using the equation: $\mathrm{lp} = \ln(\frac{p}{1-p})$, resulting in a linear predictor of -3.1833 for the medium or giant CAA model and -3.5146 for the giant CAA model. The starting value for each model was then calculated based on the minimum value of each predictor (or the maximum value of predictors with negative coefficients) and the model intercept, e.g., the starting value (α_0) for the medium or giant model was -5.7814 + 1.1211*0 + 0.0270*2 + 0.2219*3 + 2.3231*0 + 0.0075*45 - 0.1335*46 =-10.8119. The original score at the threshold for the medium or giant CAA model was then calculated as lp-α_0 =-3.1833-(-10.8119) =7.6287, resulting in a re-scaled score of 8.9698 points by multiplying the re-scaled factor 1.1758. Using the same method, the threshold score was (-3.5146-(-13.7176)) *1.8372=18.7449 points for the giant model.
